# Supplementary material for: Complexome profiling of the Chlamydomonas psb28 mutant reveals TEF5 as an early PSII assembly factor
Source: Plant Cell. 2025 Mar 17;37(6):koaf055. doi: 10.1093/plcell/koaf055 (PMC12164590; doi:10.1093/plcell/koaf055)
Supplement: koaf055_Supplementary_Data [file koaf055_supplementary_data.zip › psb28_interactive.zip!/plots/intensity/qualityPlots/peptides/FKB18_Cre10.g466850.t1.1.html]

### Cre10.g466850.t1.1

Trivial name: FKB18  
SeqCoverage [median]: 17.60  
Mass: 18.31 kDa  
  
 

| Sequence | MissedCleavage | Mass [Da] | wtIntensity | muIntensity | Score |
| --- | --- | --- | --- | --- | --- |
| GLDVVSSR | 0 | 831.45 | 0.00 | 315.40 | 66.43 |
| VEVLSVISK | 0 | 972.59 | 0.00 | 1440.86 | 59.24 |
| VYDLEEGSGAEIK | 0 | 1408.67 | 0.00 | 965.75 | 164.04 |
|  |  |  | 0.0 | 2722.0 |  |
